# Role of DNA methylation in renal cell carcinoma

**DOI:** 10.1186/s13045-015-0180-y

**Published:** 2015-07-22

**Authors:** Niraj Shenoy, Nishanth Vallumsetla, Yiyu Zou, Jose Nahun Galeas, Makardhwaj Shrivastava, Caroline Hu, Katalin Susztak, Amit Verma

**Affiliations:** Albert Einstein College of Medicine, 1300 Morris Park Avenue, Bronx, NY 10467 USA; University of Pennsylvania Perelman School of Medicine, Philadelphia, PA USA

**Keywords:** DNA methylation, Epigenetics, Kidney cancer

## Abstract

Alterations in DNA methylation are seen in cancers and have also been examined in clear cell renal cell carcinoma (ccRCC). Numerous tumor suppressor genes have been reported to be partially or completely silenced due to hypermethylation of their promoters in single-locus studies, and the use of hypomethylating agents has been shown to restore the expression of many of these genes in vitro. In particular, members of the Wnt and TGF-beta pathways, pro-apoptotic genes such as APAF-1 and negative cell-cycle regulators such as KILLIN have been shown to be epigenetically silenced in numerous studies in ccRCC. Recently, TCGA analysis of a large cohort of ccRCC samples demonstrated that aberrant hypermethylation correlated with the stage and grade in kidney cancer. Our genome-wide studies also revealed aberrant widespread hypermethylation that affected regulatory regions of the kidney genome in ccRCC. We also observed that aberrant enhancer hypermethylation was predictive of adverse prognosis in ccRCC. Recent discovery of mutations affecting epigenetic regulators reinforces the importance of these changes in the pathophysiology of ccRCC and points to the potential of epigenetic modulators in the treatment of this malignancy.

## Introduction

Renal cell carcinoma (RCC) is the most common malignant neoplasm arising in the kidney. These cancers encompass a heterogeneous group of tumors and are classified on the basis of morphology and histology into clear cell carcinomas (60 % of cases), papillary tumors, chromophobic tumors, oncocytomas, and collecting or Bellini duct tumors. The number of cases of RCC continues to rise annually and is usually more common in males (2:1) with peak incidence between 50–70 years of age. RCC is usually sporadic, but some familial forms have been reported like the one associated with von Hippel-Lindau (VHL) syndrome.

Epigenetics is the study of heritable changes in gene expression without alteration of the DNA sequence. Epigenetic changes could involve chemical modification of DNA bases via methylation of cytosines, or histone protein modification, or regulation by non-coding RNAs. DNA methylation involves the addition of a methyl group at the 5-carbon position of the cytosine ring at CpG dinucleotides within a promoter and/or enhancer and is a common reversible epigenetic alteration that has been reported in many cancers. Methylation is facilitated by DNA methyltransferase (DNMT) enzymes. Hypermethylation of promoter or enhancer CpG regions can result in inactivation of important tumor suppressor genes whereas hypomethylation of genomic DNA has been associated with chromosomal instability and tumorigenesis. The role of DNA methylation in RCC has been an active area of research over the past decade, and the various genes that are epigenetically altered in this cancer by DNA methylation have been reviewed here to give us an insight into the numerous pathways that can be influenced by hypomethylating agents.

## SFRP1 gene

It is a tumor suppressor gene encoding secreted frizzled-related protein 1 (SFRP1), a WnT antagonist controlling cell growth and neovascularization. Epigenetic silencing of SFRP1 gene occurs in premalignant tissues associated with chronic inflammation or in human cancer [1]. The tumorigenic role of SFRP1 in RCC cell lines has been demonstrated by functional analyses [2]. The evaluation of the methylation status of SFRP1 gene in clear cell RCC (ccRCC) from 10 patient-paired tissue samples using methylation-specific PCR (MSP) revealed hypermethylation in 8 of 10 tumor samples and 1 of 10 normal samples. Four ccRCC cell lines A498, 786-O, UMRC3, and ACHN revealed CGI methylation providing additional evidence of the role of methylation in the loss of SFRP1 expression in ccRCC [3].

## Rap1GAP gene

It encodes for Rap1Gap protein that inactivates Rap, which is a protein involved in cancer growth, invasion, and metastasis [4]. Rap1GAP protein is a member of the Rap GTPase-activating protein (GAP) family, which has been documented to regulate integrin-mediated cell adhesion pathways [5]. Rap1Gap promoter was found to be hypermethylated in RCC cell lines—SN12C and Caki-1—and treatment with decitabine (5-aza-2′-deoxycytidine) of SN12C cells significantly increased the relative expression of Rap1Gap. Treatment with decitabine restoring the expression of Rap1GAP showed a significant reduction in the invasiveness of SN12C and Caki1 of matrigel matrices, suggesting a role in cancer cell invasion. However, Rap1Gap was shown to have no impact on cancer cell proliferation in either of the two cell lines [6].

## KILLIN gene

KILLIN gene is involved in cell-cycle arrest and is regulated by p53. It encodes for a small nuclear DNA-binding protein that inhibits DNA synthesis in vitro and causes the S-phase arrest [7]. In a study conducted to determine the methylation and expression of KILLIN in germline, somatic, and RCC cell lines, 23 out of 41 ccRCC patients (56 %) exhibited germline methylation of KILLIN promoter whereas none of the 50 controls showed methylation (*p* < 0.0001). Somatic hypermethylation examined in 19 primary ccRCC tumor specimens and 1 metastatic RCC specimen revealed that 19 (95 %) had hypermethylation of KILLIN promoter region. In a panel of eight RCC cell lines (RC-6, RC-9, RC-13, RC-45, 786-O, ACHN, CAKI-1, and CAKI-2) and one normal kidney cell line HEK293T examined for KILLIN promoter methylation, 100 % of the RCC cell lines showed methylation in at least one of the four CpG regions identified whereas HEK293T was completely unmethylated. All the eight RCC cell lines had significant downregulation of KILLIN expression. The use of aza on seven of the eight RCC cell lines resulted in increased expression of KILLIN in six of the seven RCC cell lines [8].

## WIF-1 gene

Wnt (Wingless type) inhibitor factor-1 is a secreted glycoprotein antagonist that binds to Wnt protein. Wnt proteins are known to have a role in cell proliferation and oncogenesis [9]. Wnt antagonists are divided into two classes, namely secreted frizzled-related protein (SFRP) class and Dickkopf (Dkk) class. The SFRP family includes the SFRP-1, SFRP-2, SFRP-3, SFRP-4, SFRP-5, WIF-1, and Cerberus. These bind directly to Wnt and inhibit the signaling of the Wnt pathway whereas the Dkk family proteins bind to a component of the Wnt receptor complex to inhibit the Wnt pathway. The WIF-1 gene has been reported to have a tumor suppressor role in various cancers. A study on the role of WIF-1 as a tumor suppressor gene and the methylation status of its promoter in RCC was done using Caki-2, ACHN, and A498 RCC cell lines and a tissue microarray of 24 ccRCCs and matched normal adjacent tissues. Immunostaining for WIF-1 in normal specimens was significantly (*p* < 0.0001) higher compared with that in ccRCC specimens. The promoter region was found to be densely methylated in the three RCC cell lines and unmethylated in normal kidney samples. Treatment of the cell lines with a demethylating agent restored the gene expression significantly. WIF-1 transfection into RCC cell lines significantly reduced RCC cell viability, increased apoptotic cell fraction, and suppressed colony formation [10].

## DKK1 gene

It encodes for a protein called Dickkopf-related protein 1 which is a member of the Dickkopf family. DKK1 is a direct inhibitor of Wnt with a role in the suppression of human cancers. A study on the DKK1 methylation and expression in RCC cell lines A498 and 769-P, normal kidney cell line HK2, and RCC patient samples revealed hypermethylation of DKK1 promoter in the RCC cell lines and no methylation in the normal kidney cell line. DKK1 mRNA expression was lower in A498 and 769-P compared to that in HK2. Treatment of the RCC cell lines with azacytidine resulted in a significant increase in the expression of DKK1 and an increased percentage of apoptotic cells. Immunohistochemical study on 50 ccRCC tumor samples with corresponding 50 adjacent normal tissues revealed significantly lower DKK1 expression in RCC tissues. Transfection of DKK1 into RCC cells resulted in growth inhibition, increased apoptosis, and decreased invasion ability [11].

## SFRP2 gene

It is a tumor suppressor gene acting as a Wnt antagonist located at 4q31.3. Kawamoto et al. [12] studied epigenetic mechanisms of DNA methylation and histone modifications of the silencing of SFRP2 in RCC. They examined 20 samples from newly diagnosed RCC patients who underwent radical nephrectomy, human kidney cell line HK-2, and RCC cell lines Caki-1, Caki-2, A-498, and ACHN. By immunostaining, they noted that SFRP2 expression was strongly positive in 70 % of the normal kidney specimens whereas majority of the RCC specimens were negative. Using real-time quantitative reverse transcription (RT)-PCR, they noted that SFRP2 was expressed at a significantly higher level in HK-2 cells compared to the RCC cell lines (A-498 and ACHN showed no SFRP2 expression whereas Caki-1 and Caki-2 showed a low expression). Using bisulfite genomic sequencing, it was observed that HK-2 had no methylation of the promoter region of SFRP2 whereas Caki-2, A-498, and ACHN cell lines displayed dense methylation and Caki-1 had a lower degree of methylation compared to other RCC cell lines. Treatment with DAC restored mRNA and protein expressions of SFRP2 in all RCC cell lines.

## SFRP5 gene

This gene encodes for the protein-secreted frizzled-related protein-5(SFRP-5), a member of the SFRP family of glycoproteins that have been identified as negative modulators of the Wnt signal transduction pathway. SFRP1 and SFRP2 have been shown to be epigenetically regulated in RCC [3, 13, 14]. A study on the role of SFRP-5 and its epigenetic regulation in RCC cell lines Caki1, Caki2, ACHN, A498, and a tissue microarray consisting of 12 ccRCCs and matched adjacent tissues revealed dense methylation of SFRP-5 promoter in all RCC cell lines and ccRCC tissue samples whereas the same region in normal kidney tissue was mostly unmethylated. The expression of SFRP-5 mRNA was found to be significantly downregulated in RCC cell lines compared to normal kidney tissue (*p* < 0.01). Treatment with azacytidine restored the SFRP-5 mRNA expression suggesting its downregulation by methylation. Transfection of A498 cells with SFRP-5 resulted in reduced anchorage independent colony formation efficacy, invasive ability, and also increased apoptotic cell fraction [15].

## miR-9-1 and miR-9-3 genes

The genes miR-9-1, miR-9-2, and miR-9-3 are located on chromosomes 1, 5, and 15, respectively. These are microRNA genes encoding hsa-miR-9, a microRNA, as their product. MicroRNAs are small, non-coding RNAs which control expression by targeted suppression of gene transcription and translation. MicroRNA genes have been shown to have a role in cancer [16, 17] and can be epigenetically modified [18]. A study on the methylation status of miR-9-1 and miR-9-3 in 32 pairs of ccRCC tumor and adjacent normal tissue samples revealed significantly higher methylation levels for both (*p* = 0.00021 for miR-9-1 and *p* = 0.000074 for miR-9-3) in the tumor samples compared to their adjacent normal tissues. The methylation level was found to be nearly 2-fold higher for miR-9-1 and over 4-fold higher for miR-9-3. They also found a significant trend of increased methylation in tumor samples of higher clinical stages for both miR-9-1 (*p* = 0.024) and miR-9-3 (*p* = 0.040). The role of the methylation status of miR-9 genes in cases with metastatic recurrence was then analyzed based on follow-up information of 59 ccRCC patients, of which 21 developed metastatic recurrence. These 21 patients had a significantly higher methylation level in their primary tumors compared to those with no recurrence. There was significant greater-than-30-month-reductions in recurrence-free survival times for patients with high miR-9-1 and miR-9-3 methylation levels (*p* = 0.034 and *p* = 0.007, respectively) [19].

## GREM1 gene

The gene encodes for the protein, Gremlin 1, an inhibitor in the TGF-beta signaling pathway. GREM 1 is a secreted glycoprotein, which is an antagonist to bone morphogenetic proteins (BMP) 2, 4, and 7. This antagonistic action prevents the ligands from binding to their receptors, thereby inhibiting downstream TGF-β signaling [20]. A study investigating the methylation status of GREM1 promoter CpG islands in four RCC cell lines—SKRC1, SKRC10, SKRC52, and SKRC59; 150 tumor samples from patients with sporadic ccRCC revealed heavy methylation in the cell lines and tumor samples. There was little or no GREM1 mRNA expression in the cell lines, and treatment with 5′-aza-2-deoxycytidine induced mRNA expression in all four cell lines [21].

## BTG3 gene

This gene is located at chromosome 21q.21.1 and encodes for a protein of the BTG/Tob family, a family of structurally related proteins, which appear to have anti-proliferative activity. BTG3 has been shown to be a candidate tumor suppressor gene through its inhibitory activity on E2F1 causing negative regulation of cell cycle [22]. The methylation status of BTG3 in RCC was examined using human RCC cell lines A498, ACHN, HEK-293, normal kidney cell line HK-2, and 20 patient-matched tumor and adjacent normal tissue paired samples. The promoter region in RCC cell lines was completely methylated compared to absence of methylation in HK-2 cell line. The tumor samples were hypermethylated compared to their normal tissue samples. Real-time quantitative PCR (qPCR) analysis for expression levels showed relatively low levels of BTG3 expression in tumor samples and RCC cell lines of A498, ACHN, and HEK-293 compared to normal tissue samples and HK-2 cells. BTG3 expression was significantly increased in RCC cell lines on treatment with 5′-aza [23].

## XAF-1 gene

XIAP-associated factor-1 is a gene located at chr 17p13.1 that encodes for a protein, which is an antagonist of inhibitor of apoptosis (IAP) protein family. IAP proteins inhibit caspases which are usually active during apoptosis, and by inhibiting these IAPs, the protein encoded by the XAF-1 gene helps prevent uncontrolled growth in cancers. It is an interferon (IFN)-stimulated gene inducing high levels of XAF-1 protein in cells which are sensitive to pro-apoptotic effects of IFN-β [24]. Pre-treatment with 5-aza-dC has been shown to increase apoptosis induction with IFN in RCC cell lines, ACHN, and SK-RC-45 [25]. Of the three RCC cell lines studied—ACHN, SK-RC-45, and SK-RC-29, ACHN had the highest increase in XAF1 expression on treatment with 5-aza-dC. Transfection of siRNA against XAF1 was shown to reduce IFN-induced apoptosis in cells pretreated with 5-aza-dC, confirming XAF-1 gene’s role in apoptosis.

## APAF-1 gene

The apoptotic protease activating factor-1 (APAF-1) gene is a p53-targeted gene located at 12q23 involved in the apoptosis regulatory network. It is involved in the activation of caspase 9 which stimulates the caspase cascade leading to apoptosis. Inactivation of APAF-1 may result in failure to initiate apoptosis resulting in uncontrolled cell growth. Two studies have shown that the methylation frequency of APAF-1 gene in the tumor samples is significantly higher compared to their paired normal kidney samples and the expression of APAF-1 is significantly lower in the tumor samples [26, 27]. In the second study, two RCC cell lines (A498, ClearCa-5) were treated with sequential 5-aza-2′-deoxycytidine and significant growth inhibition was observed from the fifth day after the initial treatment. Also, the level of APAF-1 mRNA in the two RCC cell lines (A498, ClearCa-5) significantly increased after treatment with 5-aza-2′-deoxycytidine compared to pre-treatment levels, suggesting that methylation indeed inhibits the gene.

## DAPK-1 gene

Death associated protein kinase 1 gene encodes a calmodulin-dependent serine/threonine kinase involved in the cell signaling pathways of survival, apoptosis, and autophagy. It is a candidate tumor suppressor gene, which acts as a mediator of IFN-γ-induced apoptosis and its loss of expression has been linked to several tumor types. It is reported to counteract oncogene-induced transformation by activating a p19ARF/p53-dependent apoptotic checkpoint, which is designed to eliminate pre-cancerous cells during cancer development [28]. Christoph et al. [27] in their study on methylation of DAPK-1 and APAF-1 and effects of demethylating agents in kidney cancer examined 80 RCC patient samples of clear cell tumors and 20 normal kidney samples from other patients. They observed that methylation frequency of DAPK-1 was 33 % in RCC samples and 5 % in normal kidney samples.

## Nbk/Bik gene

The natural born killer gene is located at 22q13.31 and encodes for a tissue-specific BH3-only protein with pro-apoptotic properties. The pro-apoptotic activity has been reported to be dependent on Bax, a member of the Bcl-2 family [29]. In a study investigating the link between inactivation of Nbk gene and RCC, non-malignant renal tissue was observed to have significantly higher protein expression of Nbk gene on immunohistochemistry compared to weak or absent expression of Nbk in 57 ccRCC samples studied. Western blot analyses showed similar results for Nbk protein expression in RCC cell lines. RT-PCR revealed absence of Nbk mRNA in 9 of 10 RCC cell lines studied. Treatment of the cell lines with 5′-aza-2-dC resulted in increase in the expression of Nbk mRNA and protein except in one. These results indicate the role of Nbk as a tumor suppressor gene in RCC which is inactivated by promoter methylation.

## HOXB13 gene

The homeobox 13 gene located at chr 17q21.2 encodes a transcription factor that belongs to the homeobox gene family. Homeobox is a DNA sequence found within genes that are responsible for pattern of anatomical development. They encode for homeodomains which when expressed as proteins can bind to DNA and switch on cascades of other genes. HOXB13 gene has been identified to be methylated at a higher rate in primary renal tumors [30]. Seventeen (30 %) of 56 primary RCC tumor samples examined showed tumor-specific methylation of HOXB13 and none in the adjacent normal kidney tissue samples. Fifteen RCC cell lines were also studied, and 11 (73 %) of 15 showed aberrant methylation of HOXB13. Almost complete methylation of HOXB13 was detected in four cell lines while partial methylation was observed in seven others The expression of HOXB13 mRNA in the RCC cell lines was studied using RT-PCR, and the four cell lines which showed complete methylation revealed absence of the expression of HOXB13. Three of the four RCC cell lines were treated with 5-aza and TSA separately, and 5-aza alone was reported to have induced recovery of the HOXB13 expression. Transfection of KC-12 cell line with FLAG-tagged HOXB13 was done, and the re-expression of HOXB13 was reported to have shown significantly reduced number of colonies indicating its inhibitory role in colony formation. UMRC-6 RCC cells transfected with FLAG-HOXB13 showed increased caspase-3 activity, increased apoptotic activity with nuclear condensation, and cell death. HOXB13 methylation was also noted to have significant correlation to tumor grade, stage, size, and microvessel invasion. These results indicate that methylation plays an important role in the epigenetic silencing of HOXB13.

## DAL1/4.1B gene

Differentially expressed in adenocarcinoma of the lung (DAL1) gene located at chr 18p11.32 encodes for an actin-binding protein which has been reported to bind to tumor suppressor in lung cancer 1 (TSLC1) protein through its 4.1-binding motif [31]. Ten of 19 (53 %) RCC cell lines examined in a study lacked DAL1 mRNA expression [32]. Nineteen surgically resected ccRCC samples with non-cancerous renal tissues from the same patients were then analyzed, and 12 of 19 (63 %) of the primary ccRCC samples showed absent or reduced expression of DAL1 whereas the normal kidney samples showed significant expression. The methylation status of DAL1 was assessed in the 19 RCC cell lines of which 9 showed strong promoter hypermethylation. These results correlated with expression studies where hypermethylation was associated with suppressed gene expression. Caki-1, Caki-2, and KMRC-3 cell lines were treated with 5-aza-2-dC. Caki-2 and KMRC-3 cell lines, which had hypermethylated DAL1 promoter, showed re-expression of DAL1 on treatment whereas Caki-1 cell line, which lacked methylation, did not show any re-expression. These results suggest a promoter methylation causal role in DAL1 epigenetic silencing. DAL1 promoter methylation was also found to be an independent prognostic factor for metastatic recurrence in ccRCC patients.

## SPINT2/HAI2 gene

Serine peptidase inhibitor, Kunitz type 2 gene (SPINT2 is also known as Bikunin), located at 19q13.1 encodes for a transmembrane protein, which inhibits various serine proteases. The SPINT2 protein inhibits hepatocyte growth factor (HGF) activator, preventing the formation of active HGF. Active HGF has the important role of activating MET which in turn activates Ras/MAPK and PI3K/AKT effector pathways that promote RCC growth and metastasis [33]. Morris et al. [34] investigated the role of epigenetic silencing of SPINT2 in RCC. Eleven RCC-derived cell lines were subjected to treatment with 5-aza for 5 days, and 5 of 11 (SKRC39, UMRC2, 786-O, Caki-1, and KTCL26) had upregulation of SPINT2 expression. Dense promoter methylation of SPINT2 was detected in UMRC2 and SKRC39 RCC cell lines, which had shown complete silencing of SPINT2 on RT-PCR. Thirty-four of 102 RCC samples studied and only 2 of 38 normal kidney samples showed promoter region methylation. Transfection of SPINT2 gene in UMRC2 and SKRC39 cell lines resulted in reduced colony formation in in vitro colony assays. These results suggest an important role of methylation in the silencing of SPINT2 gene in RCC.

## Gamma (γ)-catenin/JUP gene

Gamma-catenin, also known as junction plakoglobin or JUP, is a cell adhesion protein and a member of the catenin family encoded by the gene located at chr 17q21. It is a common constituent of desmosomes and intermediate junctions, which are cell structures specialized for cell-to-cell adhesion. Loss of function of cell adhesion proteins like gamma-catenin has been reported to disrupt intercellular interaction between tumor cells correlating with poorer prognosis in cancers [35]. The adverse effect on prognosis of silencing γ-catenin gene expression has been reported in thyroid and lung cancers [36, 37]. The role of DNA methylation as a mechanism of silencing the γ-catenin gene in RCC was investigated in a study with 54 patient samples of primary RCC and matched normal kidney samples and 3 RCC cell lines A498, Caki-1, and Caki-2. The study revealed strong expression in normal kidney samples and weak expression in the majority of RCC tissues. The levels of mRNA transcripts were also significantly lower in RCC samples compared to normal kidney samples (*p* < 0.05). Treatment with 5-aza-dC of the three RCC cell lines (A498, Caki-1 and Caki-2) showed increased levels of γ-catenin mRNA expression after treatment compared to untreated controls.

## TCF 21 gene

Transcription factor 21 (TCF21) has been identified as a candidate tumor suppressor at 6q23-q24 that is epigenetically inactivated in many cancers [38]. The methylation status of TCF21 has been found to be very high in clear cell RCC samples compared to normal adjacent tissue samples. Treatment with DNA demethylating agent 5′-azacytidine restored part of the TCF21 expression in 786-O cell lines [39]. In another study, a significant inverse correlation was found between TCF21 methylation and expression levels in untreated renal cancer cell lines. Treatment with 1 μM or 5 μM 5-aza-dC resulted in increased expression of the gene [40].

## PCDH17 gene

PCDH17 encodes a protocadherin which is believed to have tumor-suppressing functions due to its cell adhesion, signal transduction, and growth control functions even though its exact mechanism has not been clearly defined. PCDH17 promoter region was found to be hypermethylated in 48 % of BlCa, 61 % of RCT, and 60 % of Pca samples [40].

## HIC1 gene

This gene encodes the hypermethylated-in-cancer 1 protein which functions as a transcriptional repressor. The gene has been shown to participate in complex regulatory loops regulating p53 and E2 transcription and factor 1 (E2F1) DNA damage responses [41–43]. Loss of HIC1 function has been associated with occurrence of various tumors. A study on the role of CpG island methylation of HIC1 in RCC using bisulfite conversion and pyrosequencing in 98 tumor and 70 normal adjacent tissue specimens identified HIC1 hypermethylation in tumors as an independent predictor of reduced recurrence-free survival [44].

## LRRC3B gene

Leucine-rich repeat-containing 3B (*LRRC3B*) is an evolutionarily, highly conserved leucine-rich repeat-containing protein, but its biological significance is largely unknown. It was suggested that human genome has more than 2000 LRR-containing proteins and they participate in many important processes, including plant and animal immunity, hormone–receptor interactions, cell adhesion, signal transduction, regulation of gene expression, and apoptosis. A number of microarray expression profiling studies have shown that *LRRC3B* is downregulated in various cancers suggesting that *LRRC3B* is involved in carcinogenesis [45]. In a study on its role in RCC, 57 % of the renal cancer samples studied showed hypermethylation and/or deletion. Expression analysis of LRRC3B using RT-qPCR revealed that it was almost unchanged in stages I and II of RCC but decreased 2- to 6-fold in stage III. The gene was also shown to exhibit strong cell growth inhibiting activity in colony formation experiments in vitro, further suggesting its role as a tumor suppressor gene [46].

## GATA5 gene

GATA5 belongs to a family of zinc-finger transcription factors (GATA1–6) showing high affinity to the consensus DNA sequence (A/T)GATA(A/G) [47] and is a tumor suppressor gene downregulated in various cancers. A study on the CGI methylation level of GATA5 gene in RCC cell lines RCC-GS, 786-O, A498, ACHN, RCC-HS, as well as primary renal proximal tubular epithelial cells (RPTEC) using combined bisulphite restriction analysis (COBRA) revealed that the methylation level was 77 % in 786-O, 53 % in RCC-GS, 89 % in RCC-HS, 99 % in RCC-MF, 99 % in A498, 12 % in ACHN, and 2 % in RPTEC. Increased GATA5 CGI methylation in the ccRCC group correlated with metastasis (*p* = 0.005) and decreased progression-free survival (*p* = 0.005) [48].

## RASSF1 gene

It encodes for the protein Ras-association-domain-containing protein 1 and is a tumor suppressor gene located at 3p21. The *RASSF1* gene has several major isoforms because of alternative splicing and promoter usage, but epigenetic silencing of the longer isoform, *RASSF1A*, is specifically associated with cancer [49].

RASSF1A is functionally involved in cell-cycle control, microtubule stabilization, cellular adhesion, motility, as well as apoptosis [50]. In one study, the median normalized index of methylation of RASSF1A promoter was significantly higher in papillary RCC samples compared to their normal adjacent tissue samples (2.11 vs 0.61, *p* < 0.001) [51]. Another study assessed the relation between methylation levels of RASSF1A and prognosis in 179 patients who underwent radical or partial nephrectomy for ccRCC. The level of methylation of RASSF1A promoter was found to be more in patients with stage III or IV RCC compared to patients with stage I or II (*p* = 0.043). Higher methylation level was independently associated with a poor prognosis following multivariate analysis (*p* = 0.0053) [52]. In another study, RASSF1A was observed to have significantly higher percentage of methylation in papillary RCC compared to other types of RCC [53].

## UNC5C gene

The gene product UNC5C belongs to the UNC5H functional dependence receptor family, acts as one of the Netrin-1 receptors and has the ability to induce apoptosis in the absence of the Netrin-1 ligand [54, 55]. UNC5C is expressed in the proximal tubule of the kidney where most of the RCCs originate [56]. RT-PCR study of five RCC cell lines 786-O, Os-RC-2, A498, ACHN, and Caki-1 revealed weak expression of UNC5C in Caki-1 cell line and undetectably low expression in the other four. Two out of five RCC cell lines—786-O and Os-RC2, were found to have hypermethylated UNC5C. Treatment of the two cell lines with DNA demethylation agent aza resulted in a significant increase in the expression of UNC5C. Forty-four primary RCC samples paired with their normal adjacent tissues were then studied, and all RCC samples were found to have downregulated UNC5C expression compared to their adjacent normal tissues. Of the 44, 12 showed hypermethylation of UNC5C promoter (27.3 %) in comparison to no methylation in their corresponding normal tissues.

## KRT19

KRT19 gene located at 17q21.2, encodes for the protein cytokeratin (CK) 19 [57]. It is a potential tumor suppressor that negatively regulates Akt signaling [58]. In normal kidney tissue, CK19 is expressed by distal tubules and collecting duct cells. A study was conducted on RCC cell lines 769-P, 786-O, Caki-1, Caki-2, A498, and ACHN and 112 RCC patient samples for methylation and expression levels of KRT19. The promoter methylation and mRNA relative expression levels showed a statistically significant (*p* < 0.001) inverse relation in 769-P, Caki-1, Caki-2, A498, and ACHN. The RCC cell line 786-O showed low levels of both methylation and expression. Treatment with 5 μm azacytidine of four RCC cell lines—769-P, Caki-1, Caki-2, and A498, was done (786-O and ACHN had low levels of methylation and were therefore excluded) and except for Caki-2, KRT19 promoter demethylation in three cell lines resulted in increased mRNA expression. Caki-2 had the lowest methylation levels among the four cell lines to start with. In the tissue samples, 23 out of 112 (9/52 ccRCC, 6/22 pRCC, 2/22 chrRCC, 6/16 oncocytoma) showed hypermethylation of KRT19 [59].

## GATA3 gene

Trans-acting T cell-specific transcription factor GATA-3 is a protein encoded by this gene. GATA3 protein has a role in regulating Th2 development and function and is selectively expressed during human kidney embryogenesis [60]. It regulates the expression of TβRIII (a receptor for TGF-β signaling pathway that contributes to inhibition of cell proliferation) by acting on the proximal promoter region of the TβRIII gene. GATA3 expression has been shown to be downregulated in all stages of ccRCC in two studies [61, 62], and reduced expression of GATA3 results in concomitant downregulation of TβRIII protein expression. GATA3 gene promoter has been found to be strongly methylated in tumor samples and treatment of an RCC cell line, and UMRC2 with 5′-aza resulted in dramatic increase in GATA 3 mRNA expression [61].

## TIMP3 gene

Tissue inhibitor of metalloproteinase 3 is a member of endogenous matrix metalloproteinase (MMP) inhibitors [63] and is a physiological antagonist for VEGFR-2. VEGF signaling is highly enhanced in most ccRCC tumors. A study on 105 patients who underwent radical or partial nephrectomy for ccRCC revealed that, compared to the normal renal tissue, 100 out of 105 of them had reduced TIMP3 expression [64]. Another study reported that 4 out of 12 (33 %) RCC cell lines, 28 out of 36 (78 %) tumor samples, and 0 out of 27 normal tissue samples studied had hypermethylation of TIMP3 gene promoter. The methylation was found to be similar in all grades of renal cell carcinomas suggesting that this epigenetic change might occur during early stages of cancer [65].

## TU3A gene

TU3A is a candidate tumor suppressor gene located in chromosome 3p21.1. The role of methylation in silencing of TU3A expression in human cancers like renal, bladder, cervical, breast, hepatic, ovarian, and testicular cancer cells was studied by Awakura et al. They examined the RCC cell lines of Caki-1, ACHN, and NC65 and found that ACHN had low expression of TU3A while the other two did not show any expression. The treatment with 5′-aza restored the expression of TU3A mRNA expression in all the three cell lines, and expression was even more prominent when 5′aza and Trichostatin A (histone deacetylase inhibitor) were used in combination. They also found the hypermethylation of TU3A promoter to be significantly associated with advanced tumor stage and poor disease-specific survival [66].

## FHIT gene

Fragile histidine triad protein (FHIT) is an enzyme that is encoded by the FHIT gene located at 3p14.2. The gene is reported to act as a tumor suppressor gene by growth inhibition and apoptosis induction in various cancers. The role of DNA promoter hypermethylation in the expression of FHIT in clear cell RCC was studied by Kvasha et al. [67] They conducted methylation-specific PCR for 22 paired clear cell renal cell carcinoma and non-malignant renal tissues and observed hypermethylation in 12 of 22 (54.5 %) of the ccRCC samples and almost none in the non-malignant tissues. They also noted that frequency of hypermethylation was significantly higher in patients older than 50 years than in patients younger than 50 (*p* = 0.027). They observed significantly decreased expression of FHIT in tumor samples with methylated FHIT gene using semi-quantitative RT-PCR whereas the unmethylated samples showed FHIT expression at high levels.

## DLK1 gene

The delta-like 1 homolog gene located at chr 14q32 is an imprinted gene that is paternally expressed and encodes for a transmembrane protein involved in differentiation of several cell types including adipocytes. Kawakami et al. [68] noted the common deletion at 14q32 in RCCs and hypothesized DLK1 involvement in RCC tumorigenesis. Fifteen RCC cell lines and a series of 50 cases of surgically resected primary RCC tissues (clear cell, non-papillary RCCs) and their adjacent normal kidney tissues were used in this study. They noted loss of expression of DLK1 in 39 out of 50 (78 %) primary RCC tissues whereas expression of DLK1 was maintained in every normal kidney tissue. Only 1 (NC65) of 15 RCC cell lines was noted to have expression. Using immunohistochemical analysis of DLK1 protein, they reported that it was most expressed in the renal tubular cells, which is the site of origin of RCCs. Transfection of DLK-1 resulted in increased apoptosis in all four transfected RCC cell lines compared to controls. A significant decrease in subcutaneous tumor growth in nude mice was observed after restoration of DLK1 in comparison to control cell lines suggesting tumor suppressive activity of DLK1 both in vitro and in vivo. DLK1 and GLT2 were previously reported to be reciprocally imprinted genes [69], and it was further confirmed in this study. The methylation of the CpG2 island of GLT2 gene was found to correspond with the expression of DLK1 gene. Treatment with 5′aza resulted in the recovery of DLK1 expression associated with demethylation of CpG2 region of GLT2 in cell lines.

## Connexin 32 gene

The Cx32 gene located at chr Xq13.1 encodes for a gap junction protein, which forms a part of gap junction channels that facilitate the transfer of ions and small molecules between cells. Patients undergoing long-term hemodialysis have the risk of developing acquired cystic kidney disease (ACKD), and these cysts develop from the progenitor cell of RCC, the proximal tubular cell. RCC tends to arise from the papillary adenomas that tend to arise from the cysts of ACKD. Connexin 32 has been reported to have a tumor-suppressive role against renal cell carcinoma in hemodialysis patients [70, 71]. In a study investigating the methylation status of Cx32 promoter and its role as tumor suppressor gene in RCC occurring in hemodialysis patients, 19 tumors and 19 matched non-cancerous tissues of kidneys from hemodialysis patients along with 10 matched pairs of RCC tumors and normal tissues from general patients who did not have any hemodialysis were studied. It was observed that 15 of 19 non-cancerous tissues from the hemodialysis patients were methylated and all of the 10 normal tissues of kidneys from the general patients were unmethylated which was a significant difference (*p* = 0.0001). The methylation status correlated with the expression of the gene. The mean duration of hemodialysis in patients with positive methylation status was 51.2 months whereas it was 33.6 months in patients with negative methylation status for Cx32. It was suggested that longer duration of hemodialysis induces methylation of Cx32.

## ECAD gene

ECAD, also known as E Cadherin or CDH1 or Cadherin 1 located at 16q22.1 is from the cadherin superfamily. The gene encodes for a calcium-dependent cell-cell adhesion glycoprotein. Loss of the gene function has been studied in various cancers and is thought to contribute to the progression of cancer in the later stages by increased proliferation, invasion, and/or metastasis. A good correlation has been observed between ECAD gene promoter methylation and tumor grade with grade 3 RCC tumors having 100 % methylation, grade 2 having 57 %, and grade 1 having 57 % methylation [72]. In the same study, four RCC cell lines 769-P, 786-O, Caki-1, and Caki-2 were reported to have a higher degree of methylation at most of the CpG sites of the ECAD promoter region compared to normal kidney cells which did not have any methylation. Treatment with 5-aza for 3 days resulted in increased ECAD mRNA expression in all four RCC cell lines.

## HOXA5

Homeobox A5 is a part of the homeobox genes found in cluster, named as A, B, C, and D on four separate chromosomes. This gene is part of the A cluster on chromosome 7 and codes for a DNA-binding transcription factor which controls gene expression, morphogenesis, and differentiation. The protein upregulates p53, a tumor suppressor gene, and its epigenetic silencing by methylation can thus play a significant role in tumorigenesis. A study on 62 clear cell RCC cases with paired tumor and normal adjacent tissue revealed that the methylation status of HOXA5 had a significant relationship with Fuhrman’s nuclear grade (*p* = 0.041). The high methylation status group had high Fuhrman’s nuclear grade. The HOXA5 gene had a methylation rate of 51.6 % in cancer tissue and 21 % in normal tissue. However, there was no significant relationship between the HOXA5 methylation status and patient survival [73].

## MSH2

It is located on chromosome 2 and encodes for DNA mismatch repair protein Msh2. Msh2 protein is involved in many different forms of DNA repair, including transcription coupled repair, homologous recombination, and base excision repair. Yoo et al. [73] identified MSH2 along with HOXA5 as a hypermethylated gene. At MSH2, the methylation rate was 54.8 % in cancer tissue and 26.1 % in normal tissue. There was no statistically significant relationship between the MSH2 methylation status and patient survival, similar to HOXA5. Further studies with demethylating drugs on RCC samples and cell lines could be done to identify the role of epigenetic silencing on MSH2.

## VHL gene

Von Hippel-Lindau tumor suppressor gene inactivation by promoter region hypermethylation was first reported in 1998. The VHL promoter region was reported to be hypermethylated in 11 % of the primary RCCs (11/99), and 10 of these tumors showed no evidence of concomitant VHL gene mutation suggesting that the two were mutually exclusive [74]. Similarly, epigenetic silencing of VHL gene was reported in 7 % of clear cell RCC tumors by the Cancer Genome Atlas Research Network group in 2013 [75].

## UQCRH gene

UQCRH is a tumor suppressor gene encoding a mitochondrial hinge protein. It was found to be hypermethylated and suppressed in 36 % of the clear cell RCC tumors by the Cancer Atlas Research Network group [75].

## DACH1

It is a tumor suppressor that inhibits cyclin D1 expression and is known to be suppressed in RCC by hypermethylation. Treatment with decitabine has been shown to restore the expression of DACH1 [76].

Apart from restoring the expression of the above mentioned tumor suppressor genes in RCC, hypomethylating agents may also increase the expression of a few (four known) tumor promoter genes, namely *Interleukin-8*, which promotes angiogenesis and metastasis [77]; *HLA-G*, which is known to help tumor cells evade immunosurveillance [78]; *G250*, which produces bicarbonate and neutralizes the surrounding acidic pH and aids cancer progression [79]; and *CYTIP*, which helps tumor cells evade cell death cytokines [80]. However, the overall effect of hypomethylating agents is that of tumor suppression, as a result of increased expression of a much higher number of tumor suppressor genes compared to tumor promoter genes. Hypomethylating agents have been shown to inhibit RCC cell proliferation both in vitro and in xenograft studies [81, 82] (Table 1).

**Table 1:** List of genes known to be modified by the methylation status in renal cell cancer (references in the text)

Gene	Function	Promoter methylation in renal cancer tissue compared to normal tissue	Gene expression in renal cancer tissue compared to normal tissue	Effect of hypomethylating agent on gene expression
TCF-21	Tumor suppressor gene involved in specification of differentiation	↑↑	↓	↑
PCDH17	Tumor suppressor gene involved in calcium-dependent cell adhesion	↑↑	↓	?
LRRC3B	Tumor suppressor gene involved in cell adhesion and apoptosis	↑↑	↓	?
SFRP1	Wnt antagonist tumor suppressor	↑↑↑	↓↓	?
RAP1GAP	Tumor suppressor (inactivates Rap-mediated invasion and metastasis)	↑↑	↓	↑
RASSF1	Tumor suppressor (cell-cycle control, microtubule stabilization)	↑↑↑	↓↓	↑↑
UNC5C	Tumor suppressor (induction of apoptosis)	↑↑	↓↓	↑↑
KILLIN	Tumor suppressor (cell-cycle arrest, regulated by p53)	↑↑↑	↓↓	↑↑
KRT19	Organization of myofibrils and maintaining structural integrity of epithelial cells	↑↑	↓↓	↑↑
HOXA5	Tumor suppressor (upregulates p53)	↑	?	?
MSH-2	DNA mismatch repair	↑	↓	↑
miR-9-1 and miR-9-3	Targeted suppression of gene transcription and translation	↑	↓	?
DKK	Tumor suppressor Wnt direct inhibitor	↑↑	↓	↑ (Transfection of gene inhibits cell proliferation and invasion, and increases apoptosis)
SFRP5	Tumor suppressor Wnt antagonist	↑↑↑	↓↓	↑↑ (Transfection of gene inhibits anchorage independent colony formation ability and invasion and increases apoptosis)
GATA-3	TBRIII signaling. inihibitor of cell proliferation	↑↑↑	↓↓	↑↑
TIMP-3	VEGF-3 antagonist, matrix metalloproteinase inhibitor	↑↑	↓↓	?
GREM-1 gene	TGF-β signaling inhibition	↑↑	↓↓	↑↑
WIF-1	Wnt antagonist	↑↑↑	↓↓	↑↑ (Transfection of gene reduced cell viability, suppressed colony formation, and increased apoptosis)
UCHL1	Ubiquitination of proteins	↑↑	↓	↑
BTG3	E2F1 inhibition → negative regulation of cell cycle	↑↑↑	↓↓	↑↑
TU3A	Tumor suppressor, mechanism unknown	↑↑	↓	↑
14-3-3 sigma	Causes G2 phase block for DNA repair	↑	↓	↑
p16	Cyclin kinase-dependent inhibition → causes cell-cycle arrest	↑	↓	?
SFRP2	Wnt antagonist	↑↑↑	↓↓	↑↑
FHIT	Tumor suppressor, induction of apoptosis	↑↑	↓↓	?
XAF-1	Pro-apoptotic gene, inhibitor of “inhibition of apoptosis” protein	↑↑	↓	↑
APAF 1	Pro-apoptotic gene, activation of caspase 9	↑↑↑↑	↓↓↓	↑↑
DAPK-1	Mediation of IFN-γ induced apoptosis	↑	↓	?
DLC-1	Inhibitor of RhoAGTPase	↑	↓	?
DLK-1	Pro-apoptotic gene	↑↑	↓↓	↑↑ (Transfection decreases cell proliferation, increases cell apoptosis)
HOX-B-13	Pro-apoptotic gene	↑↑↑	↓↓	↑↑ (Transfection decreases cell proliferation)
DAL-1	Protein 4.1-related tumor suppressor gene	↑↑	↓	↑
SPINT-2	Tumor suppressor: inhibits HGF which activates Ras pathway	↑↑	↓	? (Transfection decreases cell proliferation)
Gamma-catenin	Involved in cell-cell adhesion. Loss of function correlates with poorer prognosis in cancer	↑↑↑	↓↓	↑↑
Connexin-32	Gap junction protein. May be involved in the increased incidence of RCC in maintenance hemodialysis patients	↑↑	↓↓	?
Ecad	Tumor suppressor: encodes for cell-cell adhesion glycoprotein	↑↑	↓↓	↑↑
HLA-G	Tumor promoter: involved in helping tumor cells evade immunosurveillance	?	↑	↑
IL-8	Tumor promoter: promotes angiogenesis and metastasis	↓ (genomic hypomethylation)	↑	?
CA9/G250	Tumor promoter: expressed in hypoxic tumors, produces bicarbonate which neutralizes the surrounding acidic pH and aids cancer progression	↓↓	↑↑	↑
CYTIP	Promotes metastasis. Helps tumor cells evade cell death cytokines	↑↑	?	↑

## Genome-wide studies reveal novel epigenomic alterations in RCC that affect regulatory regions of the genome

Even though recent studies have shown that epigenetic changes at enhancers can influence carcinogenesis, most methylomic studies in RCC detailed above focused on changes at promoters and were single locus studies. We studied genome-wide patterns of DNA methylation in ccRCC at a high resolution analysis of 1.3 million CpGs with the HELP assay. Microdissected renal tubular cells were used as controls. We observed that the RCC samples were characterized by aberrant hypermethylation that preferentially affected gene bodies. The hypermethylation was particularly enriched in kidney-specific enhancer regions associated with H3K4Me1 marks. This genome-wide hypermethylation in ccRCC is in contrast to other solid tumors such as esophageal cancer, hepatocellular cancer, head and neck cancers, etc., that are characterized by genome-wide hypomethylation. MOTIF analysis of aberrantly hypermethylated regions revealed enrichment for binding sites of AP2a, AHR, HAIRY, ARNT, and HIF1 transcription factors, reflecting possible contributions of dysregulated hypoxia signaling pathways to epigenetic changes in RCC. The functional importance of this aberrant hypermethylation was demonstrated by selective sensitivity of RCC cells to low levels of DNA methyltransferase inhibitors. Most importantly, methylation of enhancers was predictive of adverse prognosis in a large cohort of ccRCC samples [83].

The Cancer Genome Atlas Research Network group also reported that increasing promoter hypermethylation frequency correlated with higher stage and grade in clear cell RCC [75]. Apart from discovering hypermethylation of VHL and UQCRH tumor suppressor genes in some ccRCC samples (as described above), they also made an interesting evaluation of the global consequence of mutations in specific epigenetic modifiers. Inactivating mutations of the SetD2 gene occur in a few cases of ccRCC and was found to be associated with increased loss of DNA methylation. SetD2 is a H3K36 methyltransferase. H3K36 trimethylation has been suggested to be involved in the maintenance of the heterochromatin state. DNA methyltransferase 3A (DNMT3A) binds to trimethylated H3K36 and methylates nearby DNA resulting in transcriptional silencing. Consequently, it was thought that reduction in trimethylated H3K36 levels through SetD2 inactivation could lead to regional loss of DNA methylation [75]. It would be interesting to study the effect of hypomethylating agents in SetD2 mutant and wild-type ccRCC to determine if inactivating mutations of SetD2 can be indicators of poor response to hypomethylating agents.

## Conclusions

DNA methylation plays an important role in the development and progression of renal cell carcinoma. Epigenetic silencing of numerous tumor suppressor genes caused by hypermethylation of enhancer and promoter CpGs leads to increased tumor cell proliferation, invasion, and metastasis. In particular, activation of Wnt pathway by suppression of Wnt antagonists, namely SFRP1, SFRP2, SFRP5, and WIF-1 appears to play a very prominent role in the proliferation of many RCC cell lines and patient tumor samples—as determined by a higher percentage of methylation in cancer tissue compared to normal tissue, very low expression compared to normal tissue, significant inhibition of proliferation, and increase in apoptosis caused by transfection of the Wnt antagonist genes. Similarly, TGF-β pathway activation by promoter methylation-induced inhibition of pathway inhibitory genes GATA-3, GREM-1, and SMAD-6 plays a significant role in many RCC cell lines. Silencing of pro-apoptotic genes such as APAF-1 and negative regulators of cell cycle such as KILLIN and RASSF1 also appear to play an important role.

Hypomethylating agents have shown inhibition of renal carcinoma cell proliferation both in vitro and in xenograft studies by the re-expression of numerous tumor suppressor genes silenced by enhancer/promoter hypermethylation [81, 82]. Despite the possibility of simultaneous increased expression of some tumor promoter genes, the overall effect of hypomethylating agents on RCC is that of tumor suppression due to increased expression of a much higher number of tumor suppressor genes. The genome-wide hypermethylation in RCC is in contrast to other solid tumors which demonstrate widespread hypomethylation, thereby making hypomethylating agents and other epigenetic modulators potential therapeutic agents in RCC, along with emerging targeted therapies [84–86].
